# An Analysis of Trabecular Bone Structure Based on Principal Stress Trajectory

**DOI:** 10.3390/bioengineering10101224

**Published:** 2023-10-20

**Authors:** Jiwu Zhang, Haoran Li, Yuqing Zhou, Songhao Chen, Qiguo Rong

**Affiliations:** Department of Mechanics and Engineering Science, College of Engineering, Peking University, Beijing 100871, China; zjw3249874@163.com (J.Z.); 2001111706@pku.edu.cn (H.L.); yuqingzhou@stu.pku.edu.cn (Y.Z.); lijfuj@stu.pku.edu.cn (S.C.)

**Keywords:** trabecular bone, finite element analysis, stress visualization, principal stress trajectory

## Abstract

To understand the mechanism of Wolff’s law, a finite element analysis was performed for a human proximal femur, and the principal stress trajectories of the femur were extracted using the principal stress visualization method. The mechanism of Wolff’s law was evaluated theoretically based on the distribution of the principal stress trajectories. Due to the dynamics of the loads, there was no one-to-one correspondence between the stress trajectories of the fixed load and the trabeculae in the cancellous architecture of the real bone. The trabeculae in the cancellous bone were influenced by the magnitude of the principal stress trajectory. Equivalent principal stress trajectories suitable for different load changes were proposed through the change in load cycle and compared with the anatomical structure of the femur. In addition, the three-dimensional distribution of the femoral principal stress trajectory was established, and the adaptability potential of each load was discussed. The principal stress visualization method could also be applied to bionic structure design.

## 1. Introduction

Bone is a natural biocomposite composed of the cortex and trabecular (cancellous) structure [1]. The functional relationship of the trabecular structure in the body is a complex mechanical biological characteristic, resulting from bone self-adaptation optimization according to the mechanical load state [2]. Therefore, the trabecular bone forms a structure-specific arrangement according to the external load’s size and direction, which play particular roles in the supporting performance of the bone. Studies have shown that trabecular bone has excellent effects on the load transmission and energy absorption of major joints (such as knees, hips, and spine) [3]. 

As an initial study on the internal bone architecture, Meyer and Wolff et al. [4,5,6] found that the arrangement of bone trabeculae formed the optimal performance effect, changing the intensity and area of the external load. Therefore, “Wolff’s law” with structure and function as the main content was proposed, which opened the way to study trabeculae’s structural morphology. Cater et al. [7] reconstructed frozen trabecular sections using the tissue section technique and calculated trabeculae’s morphological parameters. This method could be used to describe the trabecular structure with morphological parameters. After that, Whitehouse and Dyson [8] obtained trabecular bone images using scanning electron microscopy with the same principle as the slicing technique. The measurement methods of trabecular circumference and area and the structural state of trabeculae were analyzed. A qualitative description of trabeculae such as “good connectivity” and “unconnected” was proposed, laying a foundation for the morphological parameters’ follow-up study. The direction and connectivity of trabeculae directly affect the mechanical properties of trabeculae [9]. In addition, the two main types of bone trabeculae, plate and beam, play essential roles in determining the apparent strength and failure behavior of bone trabeculae. However, trabecular bone is the internal tissue structure at the long bone ends, wrapped with the compact bone outside. Because of the complex connection of the trabecular structure, it is difficult to obtain the complete trabecular bone structure with experimental measurements. Medical imaging equipment such as Micro-CT could obtain clear and complete trabecular bone images. On this basis, morphological parameters could be used to quantitatively describe the trabecular bone structure to measure and evaluate the morphological characteristics of the trabecular bone structure [10]. 

The finite element model of trabecular bone was usually assumed to be an isotropic homogeneous organization [11,12]. Recent studies have emphasized the shortcomings of this hypothesis. They demonstrated the influence of heterogeneity on biomechanical models’ results for predicting the elastic modulus of trabecular bone tissue. Micro-finite element (micro-FE) analysis has become a popular tool for determining trabecular bones’ mechanical properties. Boyle [13] used Micro-CT to establish a topology optimization approach to create natural optimal structures under daily walking loads. The structure qualitatively revealed several anisotropic trabecular regions comparable to native human femurs. From a quantitative point of view, the calculated bone volume fractions in various regions were consistent with a quantitative CT analysis. In addition, Adachi [14] used a large-scale voxel finite element model to perform computer simulations of human proximal femoral trabecular remodeling, verifying the mechanical mechanism of Wolff’s law. A three-dimensional reconstruction of trabecular bone created from high-resolution cross-sectional images is often used as the basis for micro-FE geometry. The most common way of creating a model is to convert voxels into brick elements of the same shape [15,16,17]. The simulation of micro-FE can only be achieved by using computationally efficient algorithms and parallel computing [18]. Computational bone remodeling requires a large amount of finite element analyses in an iterative manner and other optimization calculations. 

Furthermore, trabecular bone is considered to be structurally optimized where it adapts to long-term loading by controlling its density and internal structure [19]. Boyle [13] used a topology optimization method to perform a micro-level, three-dimensional finite element bone remodeling simulation on the human proximal femur and analyze the results to determine the validity of Wolff’s hypothesis. Huo [20] coupled the Physiological Stochasticity in Bone Remodeling into the conventional Topology Optimization algorithm to predict the cancellous structure of the human femur. Goda [21] adopted a multi-scale topology optimization method to achieve the optimal distribution of bone trabecular structures for the proximal femur under physiological loads and combined the spatial arrangement characteristics of natural bone trabecular structures. However, topology optimization methods require enormous computational resources, and each optimization iteration requires repeated finite element simulations. Its complexity increases with the increase in the global model size and dimension expansion.

Therefore, some studies are limited to the region of interest (ROI) of the proximal femur. Kim [22] quantitatively verified the accuracy and efficiency of the local bone microstructure reconstruction method based on topology optimization. By quantitatively analyzing the load dependence of the trabecular microstructure in the ROI of the proximal femur, the spatial characteristics under different load conditions were analyzed [23]. However, based on the ROI, it was not possible to consider the mapping relationship between external mechanical load stimuli and the spatial structural characteristics of bone trabeculae, which is a fundamental assumption of Wolff’s law. The common feature of these various numerical methods is derived from the experience of precisely stimulating the bone remodeling process. These phenomenological methods can predict the progress of bone remodeling. However, they do not explain the mechanism of the formation of trabecular structures, which is the basic assumption of Wolff’s law.

In this paper, a trabecular bone structure analysis method based on principal stress locus was proposed. Firstly, a finite element model of the femur was established, and the stress distribution results of different loads were thus obtained considering the force change in the femur load. The principal stress trajectory of the femur was extracted using the principal stress visualization method. According to the femur’s load change and the distribution of the principal stress trajectory related to kinematics, the equivalent principal stress distribution suitable for different load changes was proposed through the load cycle change. The mapping relationship between external mechanical load stimuli and the spatial structural features of bone trabeculae can be intuitively established through the principal stress trajectory to improve the understanding of spatial features under various load conditions. In addition, it helps to establish a clinically applicable and reliable structural optimization prosthesis according to the principal stress trajectory. This method builds a biomimetic-optimized porous structure prosthesis based on stress field distribution, which is simpler, more efficient, and direct compared to topology optimization methods.

## 2. Materials and Methods

### 2.1. Modeling and Simulation Conditions

This paper used the finite element method to simulate the gait-loading scene of multiaxial force combination [24]. The geometric shape of the standardized left femur was used for analysis [25]. The two-dimensional femoral shape on the standardized femur’s coronal plane was used as the finite element model to simplify the calculation process. 

**Figure 1:**
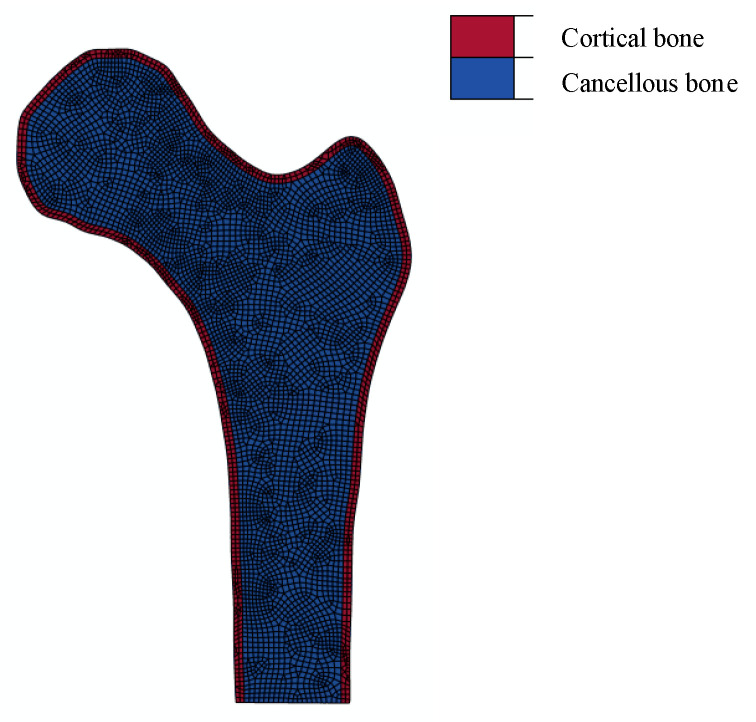
Finite element model of the proximal femur.

A finite element subroutine for calculating the magnitude and direction of the principal stress was deployed in the material subroutine UMAT of the commercial finite element software package ABAQUS/Standard. As shown in Figure 1, the model was divided into the cortical bone and cancellous bone. The thickness of the cortical bone was 2 mm. 

According to the literature [26], the isotropic simplification does not show much difference from the orthotropic material property assignment. Therefore, all the materials in this study were considered to be isotropic and homogeneous. The material properties of the models were assigned on the basis of previous studies (Table 1) [27]. The cortical bone and cancellous bone were meshed with triangular elements, as these elements were well-suited to mesh irregular and complex geometries. Aimed at the satisfying convergence of the numerical results, the meshes of the models were dense enough, as shown in Figure 1. The numbers of elements and nodes are shown in Table 2.

**Figure 2:**
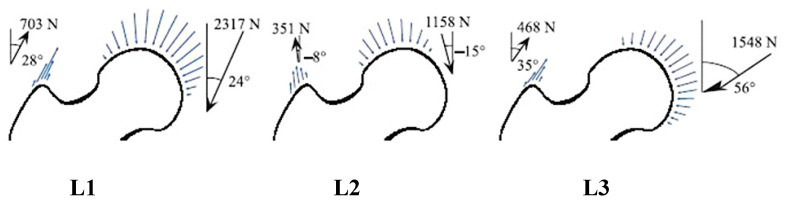
External loading conditions applied to the hip joint. L1: one-legged stance; L2: extreme ranges of motion of abduction; and L3: adduction.

As a representative daily loading condition, three loading cases of one-legged stance (L1), extreme ranges of motion of abduction (L2), and adduction (L3) were assumed [28]. These external loadings were applied to the joint surface and the greater trochanter (Figure 2). The lower boundary that corresponded to the diaphysis was fixed. The simulation results were discussed only for the proximal region of the finite element model to neglect an artificial influence of the fixed boundary condition. 

### 2.2. Visualization Method

Since the conventional finite element model cannot provide the distribution of the principal stress trajectory, it was necessary to calculate the principal stress trajectory using the finite element. 

In this paper, the fourth-order Runge–Kutta method of evenly-spaced streamline provided by the Paraview software (version 5.6) was used to calculate the principal stress trajectory [29]. 

A principal stress trajectory is a curve everywhere tangent to the flow,

(1) dρ(τ)/dt=v(ρ(t))

where ρ(τ) is a point along the streamline and v(ρ(t)) is the flow vector at the point. Given a seed with τ=0, the streamline is obtained by solving the above differential equation step by step:

(2) $$\rho (\tau +\Delta \tau )=\rho (\tau )+\int_{\tau }^{\tau +\Delta \tau }v(\rho (\tau ))d\tau$$

The steps of RK4, a well-known numerical integrator, are:

(3) $$\rho (\tau +s)=\rho (\tau )+\frac{1}{6}\left(\Delta \rho_{1}+\Delta \rho_{2}+\Delta \rho_{3}+\Delta \rho_{4}\right)$$

(4) $$\Delta \rho_{1}=s\times v(\rho (\tau ))$$

(5) $$\Delta \rho_{2}=s\times v\left(\rho (\tau )+\frac{\Delta \rho_{1}}{2}\right)$$

(6) $$\Delta \rho_{3}=s\times v\left(\rho (\tau )+\frac{\Delta \rho_{2}}{2}\right)$$

(7) $$\Delta \rho_{4}=s\times v\left(\rho (\tau )+\frac{\Delta \rho_{3}}{2}\right)$$

where $\rho \left(\tau_{n}\right)$ is the current integration position, s is the selected step length, $\rho \left(\tau_{n+1}\right)$ and the position of the next point are obtained via integration, and $\upsilon \left(\rho \left(\tau \right)\right)$ is the tangent direction of the point. 

To reduce the error as much as possible and make the step size as large as possible, D. Stalling [30] proposed an adaptive step size mechanism based on local truncation error.

(8) $$\varepsilon =\frac{\Delta \rho_{4}}{6}-6(\rho (\tau +s))\times \frac{s}{6}$$

(9) $$s=\left\{\begin{array}{l} 2s,\;\;\;\;\;\;\;\;\;\varepsilon \leq \varepsilon_{min}\\ s,\;\;\;\;\varepsilon_{min}<\varepsilon <\varepsilon_{max}\\ 0.5s,\;\;\;\;\;\;\varepsilon <\varepsilon_{max} \end{array}\right.$$

Given an error tolerance range $\left(\varepsilon_{min},\varepsilon_{max}\right)$, the step size is doubled when $\varepsilon <\varepsilon_{min}$ and halved when $\varepsilon >\varepsilon_{max}$.

Due to the adaptive step size, the distance between two successive points obtained, $\rho \left(\tau_{n}\right)$ and $\rho \left(\tau_{n+1}\right)$, is not fixed. To create evenly spaced samples for distance control, we use cubic Hermite polynomial interpolation to sample each streamline:

(10) $$\Phi (\mu )=A\mu^{3}+B\mu^{2}+C\mu +D;\mu =\left(\tau_{s}-\tau_{n}\right)/\left(\tau_{n+1}-\tau_{n}\right)\in [0,1]$$

where $\tau_{n}$ and $\tau_{n+1}\;$are the curve lengths for points $\rho \left(\tau_{n}\right)$ and $\rho \left(\tau_{n+1}\right)$ respectively. $\tau_{s}\in \left[\tau_{n},\tau_{n+1}\right]$ is the curve length for an evenly interpolated sample:

(11) $$\begin{array}{l} A=2\Phi \left(0\right)-2\Phi (1)+\Phi^{\prime }(0)+\Phi^{\prime }(1);\;\;\;\;\;\;\;\;\;\;\;\;\;\;C=\Phi^{\prime }(0)\\ B=-3\Phi \left(0\right)+3\Phi \left(1\right)-2\Phi^{\prime }\left(0\right)-\Phi^{\prime }(1);\;\;\;\;\;\;\;\;D=\Phi (0) \end{array}$$

with boundary conditions:

(12) $$\begin{array}{l} \Phi (0)=\rho \left(\tau_{n}\right);\;\;\;\;\Phi^{\prime }(0)=\left(\tau_{n+1}-\tau_{n}\right)v\left(\rho \left(\tau_{n}\right)\right)\\ \Phi (1)=\rho \left(\tau_{n+1}\right);\;\Phi^{\prime }(1)=\left(\tau_{n+1}-\tau_{n}\right)v\left(\rho \left(\tau_{n+1}\right)\right) \end{array}$$

where $\tau_{s}$ is initialized to a given sampling size ζ and is incremented by ζ following each sample interpolation that occurs when $\tau_{s}$ falls within $\left[\tau_{n},\tau_{n+1}\right]$. The interval of two successive points, i.e., the step size $\tau_{n}-\tau_{n+1}$, may be tens of times the field cell size; hence, there may be many evenly spaced samples in the range, which can be quickly generated using forward difference equations:

(13) $$\begin{array}{c} \Phi_{1}\left(\mu \right)=\Phi \left(\mu +\mu_{8}\right)-\Phi (\mu )=3A\mu_{6}\mu^{2}+\left(3A\mu_{5}^{2}+2B\mu_{3}\right)\mu \\ \;+A\mu_{6}^{3}+B\mu_{s}^{2}+C\mu_{6}\\ \Phi_{2}\left(\mu \right)=\Phi_{1}\left(\mu +\mu_{6}\right)-\Phi_{1}(\mu )=6A\mu_{6}^{2}\mu +6A\mu_{6}^{3}+2B\mu_{6}^{2}\\ \Phi_{3}\left(\mu \right)=\Phi_{2}\left(\mu +\mu_{6}\right)-\Phi_{2}(\mu )=6A\mu_{6}^{3}=\mathrm{constant} \end{array}$$

where $\mu_{\delta }=\zeta /\left(\tau_{n+1}-\tau_{n}\right)$. Once $\Phi_{1}\left(\mu \right)$, $\Phi_{2}\left(\mu \right)$, and $\Phi_{3}\left(\mu \right)$ are computed for the first evenly spaced sample within an interpolation interval, the subsequent samples within the same interval can be recursively obtained using three additions per sample:

(14) $$\begin{array}{l} \Phi \left(\mu_{k+1}\right)=\Phi \left(\mu_{k}\right)+\Phi_{1}\left(\mu_{k}\right)\\ \Phi_{1}\left(\mu_{k+1}\right)=\Phi_{1}\left(\mu_{k}\right)+\Phi_{2}\left(\mu_{k}\right)\\ \Phi_{2}\left(\mu_{k+1}\right)=\Phi_{2}\left(\mu_{k}\right)+\Phi_{3}\left(\mu_{k}\right) \end{array}$$

where $\Phi \left(\mu_{k}\right)$ and $\Phi \left(\mu_{k+1}\right)$ are evenly interpolated samples k and k+1 within the same interval, respectively. In order to verify the effectiveness of this method, the distribution of the principal stress trajectory of the models (cantilever beam structure and X-shaped structure) was calculated and compared with the results of the literature (see Appendix A).

### 2.3. Equivalent Principal Stress Trajectory Distribution

Three load cases represent daily activities: one-legged stance (6000 cycles per day), extreme ranges of motion of abduction (2000 cycles per day), and adduction (2000 cycles per day), and the three loads are given at a ratio of 3:1:1 [18,28,31]. Therefore, the weighted summation of different principal stress direction fields and stress fields generated by adult men’s average load history during walking was carried out:

(15) φ=0.6×φ1+0.2×φ2+0.2×φ3

where *φ* is the direction of the principal stress, *φ*1, *φ*2, and *φ*3 are the directions of the principal stress, L1, L2, and L3, respectively. The weight coefficient distribution is based on the frequency of three kinds of loads in daily activities.

## 3. Results

### 3.1. Stress Distribution

**Figure 3:**
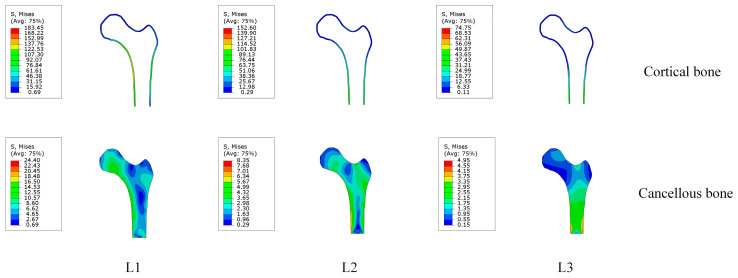
The von Mises stress of the proximal femur. L1: one-legged stance; L2: extreme ranges of motion of abduction; and L3: adduction.

The trabecular bone stress distribution change under a single loading was obtained depending on the loading case. The stress of all the models was compared in our study (Figure 3 and Table 3).

The maximum von Mises stress of the cortical bone in loading condition L1 was 20.20% larger than that in loading condition L2. In comparison, the stress in loading condition L2 was 104.14% larger than that in loading condition L3. The high-stress area of cortical bone was concentrated on the inner and outer sides of the femur. The medial side extends from the femoral head to the distal end along the medial cortex; the lateral extension extends from the high-stress zone of the lateral wall of the lateral femoral crest to the distal end. The inner part forms an oblique support of the proximal femur cantilever structure, reducing the bending stress and deflection of the femur; the lateral part can effectively reduce the sliding and deviation of the femoral neck under a physiological load. 

As shown in Figure 3, the maximum von Mises stress of the cancellous bone in loading condition L1 was 24.04 MPa, 192.21% larger than that in loading condition L2 and 392.93% larger than that in loading condition L3 (Table 3). Figure 3 shows that the highest von Mises stress were concentrated in the greater trochanter and femoral neck. The von Mises stress distribution of all three loading cases was similar.

### 3.2. 2D Principal Stress Trajectory Distribution

**Figure 4:**
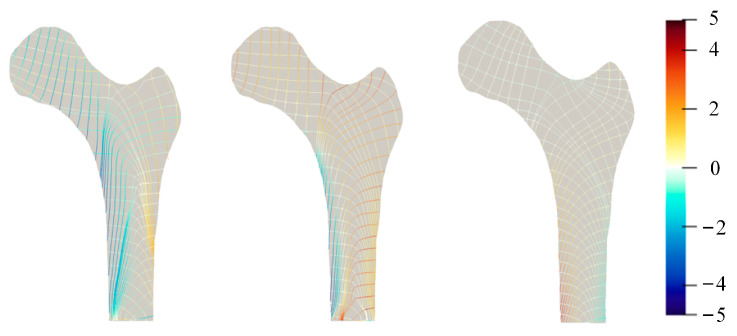
The principal stress trajectory of the proximal femur. L1: one-legged stance; L2: extreme ranges of motion of abduction; and L3: adduction.

In this section, the principal stress trajectory for different loads was calculated using the principal stress magnitude and direction. The principal stress value was indicated by the color scale of the stress trajectory, which assigned tensile stress to red and compressive stress to blue (Figure 4). The magnitude of the principal stress was specified using the color saturation, and the minimum amount of the principal stress was associated with the minimum color saturation. The trajectory in the area with a zero stress value presents a background color. As shown in Figure 4, different stress trajectory distribution trends were shown in the femur’s various regions. In the coronal section, the trabecular bone is orientated in the direction of the compressive load applied to the articular surface in the femoral head, and orientated in the abduction tensile direction load in the greater trochanter. In the femoral neck, an orthogonal pattern of the trabecular bone forms in the biaxial compression and tension load state under the bending load. Depending on the load, the distribution of the principal stress trajectory varies slightly. The compressed region for load L1 is larger than the tensile region. In comparison, the tensile region for load L2 is marginally larger than the compressed region and the tensile compression region for load L3 is almost symmetrical. Simultaneously, the direction and size of the stress trajectory in the different regions also differ slightly.

**Figure 5:**
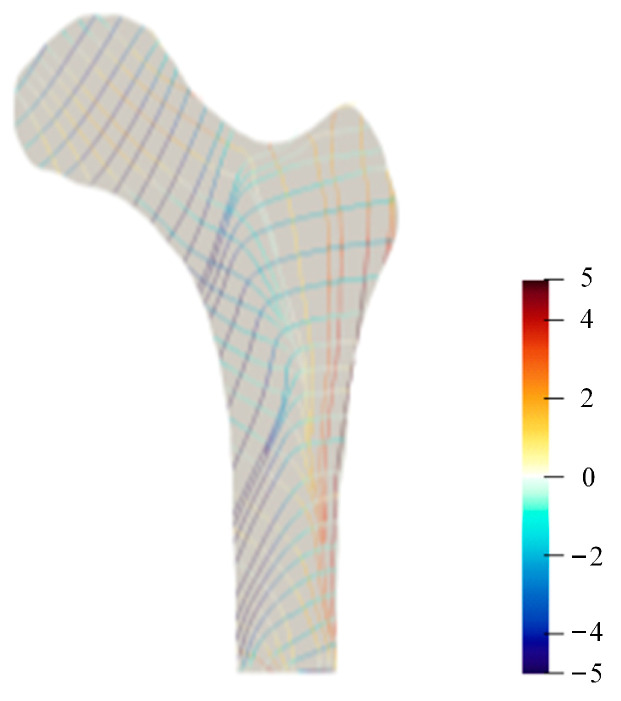
The equivalent principal stress trajectory of the proximal femur.

Figure 5 shows the equivalent principal stress trajectory distribution obtained by the three stress trajectory distributions. 

### 3.3. Three-Dimensional Principal Stress Trajectory Distribution

**Figure 6:**
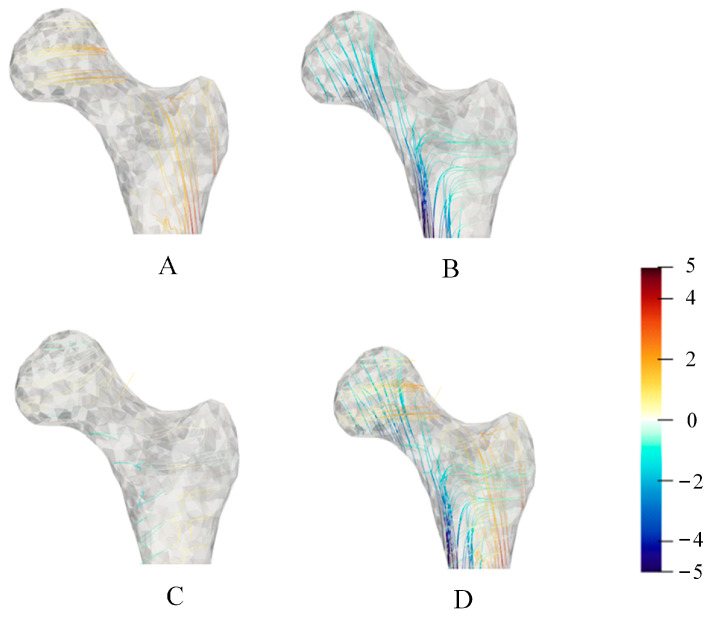
Three-dimensional principal stress trajectory of the proximal femur. (**A**) First principal stress trajectory; (**B**) second principal stress trajectory; (**C**) third principal stress trajectory; and (**D**) all principal stress trajectory.

**Figure 7:**
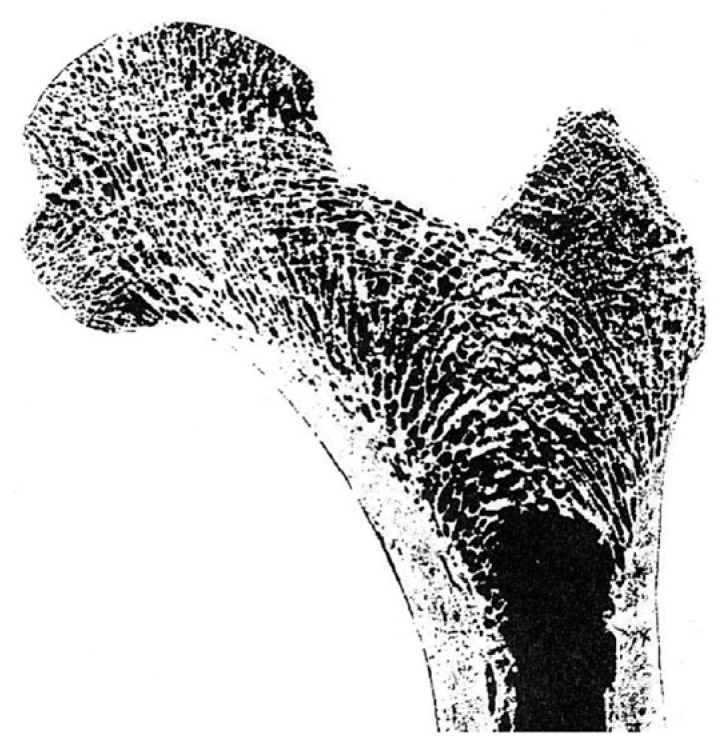
The coronal cross-section of an actual femur (right) from Wolff.

To describe the distribution of the principal stress trajectory in the femoral stress state more clearly, the three-dimensional principal stress trajectory distribution was also drawn using the above method. As shown in Figure 6, this method could also clearly show the three-dimensional femoral principal stress trajectory distribution. The principal stress trajectory distribution appeared similar to that observed in the cross-sectional photograph of the human proximal femur (Figure 6 and Figure 7).

## 4. Discussion

In this study, the principal stress trajectory distribution in trabecular bone was applied according to the principal stress distribution and compared with different load cases. The change in gait load can change the distribution of the femoral principal stress trajectory. The results showed that the equivalent principal stress trajectory could correspond to the anatomical structure through different load distributions. At the same time, the trabecular structure not only follows the direction of the principal stress trajectory, but also corresponds to the anatomical structure of the trabecular bone. The three-dimensional bone structure can correspond well to the anatomical structure by using the two-dimensional principal stress trajectory extraction method. 

As shown in Figure 3, the von Mises stress is mainly concentrated in the cortical bone, which means that the cortical bone bears most of the load. This is the reason why the cortical bone is denser than the cancellous bone. For the cancellous bone, the maximum Mises stress is concentrated in the left side of the femur, close to the results of previous studies [32]. For load 1, the Mises stresses are mainly concentrated on the left side and the upper part. For load 2, the Mises stresses are focused on the left and upper side and the right side. For load 3, the Mises stresses are mainly concentrated at the lower end of the femur. The three loads reveal that the stress distribution in the femur is always a changing process during movement.

Based on the stress distribution calculated by the finite element, three principal stress trajectories are obtained by using the fourth-order Runge–Kutta method of evenly spaced streamline and assigned different values depending on the magnitude of the principal stress values. To obtain the distribution of the femoral trabecular structure’s principal stress trajectory closer to the real situation, the equivalent stress trajectory was obtained by weighting the sum of the three loads. The equivalent principal stress trajectory direction can represent the trabecular structure’s growth direction in the femur. Moreover, the absolute value of the principal stress on the trace line is approximately zero, which corresponds to the cavity position of the femoral structure. It is consistent with Roux’s concept of the functional adaptation of osseous tissue [33]. 

Some have argued that Wolff’s law does not reveal the structural properties of bone trabeculae. Cowin [34] proposed that the trabeculae of cancellous bone embody the stress trajectories determined from the stress analysis of a homogeneous and continuous elastic object of the same shape as the bone and loaded in the same way. Stress trajectories and trabecular structures cannot be correlated in the way suggested by this premise. However, the fact is that the femur’s loading is a dynamic process, and it cannot be strictly required that the femur model’s principal stress trajectories correspond to each other and the real anatomical structure. It is also proposed that the principal stress trajectory is mutually orthogonal, but trabecular structures are not mutually orthogonal. This is also due to the variation in the femoral loading, resulting in an equivalent principal stress trajectory structure formed by the principal stress trajectory’s interaction under multiple loads, which does not necessarily satisfy mutual orthogonality.

Due to the limitations of the two-dimensional model, the three-dimensional femur model’s principal stress trajectory was extracted to analyze the relationship between the principal stress trajectory and the trabecular structure and discuss the Wolff’s law’s validity. As shown in Figure 6 and Figure 7, the distribution of the principal stress trajectory corresponds to the direction of the femur’s trabecular structure, and the sparseness of the trabeculae correlates with the absolute magnitude of the stress values on the principal stress trajectory. This paper also describes the distribution of the second principal stress trajectory, which rotates mainly in the femur’s axial position. This is due to a certain amount of torsion of the femur during the force’s motion, thus resulting in a rotational trace around the femur in the second principal stress trajectory. 

By showing the distribution of the principal stress trajectory in two and three dimensions, it was revealed that the trabecular structures grow along the direction of the principal stress trajectory and are influenced by the magnitude of the principal stress values. 

Since the value of the principal stress at the trabecular structure’s corresponding location is as small as close to zero, no trabecular structure is stimulated at that location. Therefore, the trabecular structure is not only dependent on the direction of the stress trajectory, but is also influenced by the magnitude of the principal stress trajectory.

## 5. Conclusions

The stress visualization method was used to extract the principal stress trajectory from the femoral structure. The equivalent principal stress trajectory distribution was established and compared to the anatomical structure according to the motion load change, which further elaborates and illustrates Wolff’s law. The trabecular structure follows the direction of the principal stress trajectory, but is also influenced by the magnitude of the principal stresses. Simultaneously, a three-dimensional principal stress trajectory distribution method was developed to describe the distribution of the stress trajectory. Compared with topological optimization methods for describing the spatial structural characteristics of bone trabeculae, the principal stress trajectory method can easily and intuitively establish the mapping relationship between external loads and the bone trabeculae structure. This description of the three-dimensional principal stress trajectory provides new ideas for establishing a bionic optimized porous prosthesis structure.

## Figures and Tables

**Table 1:** Material properties in the FEA models.

Model	Young’s Modulus (MPa)	Poisson’s Ratio
Cortical bone	10,500	0.3
Cancellous bone	150	0.3

**Table 2:** Number of elements and nodes in the FEA models.

	Elements	Nodes
Cortical bone	2313	454
Cancellous bone	1553	490

**Table 3:** Maximum von Mises stress (MPa).

	L1	L2	L3
Cortical bone	183.45	152.60	74.75
Cancellous bone	24.40	8.35	4.95

**Figure A1:**
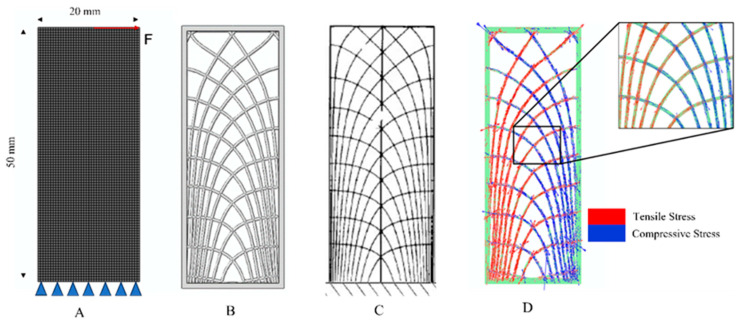
Cantilever beam model. (**A**) Finite element model of the cantilever beam; (**B**) the principal stress trajectory of cantilever beam; (**C**) the principal stress trajectory from Cowin; and (**D**) the principal stress distribution of porous structure.

**Figure A2:**
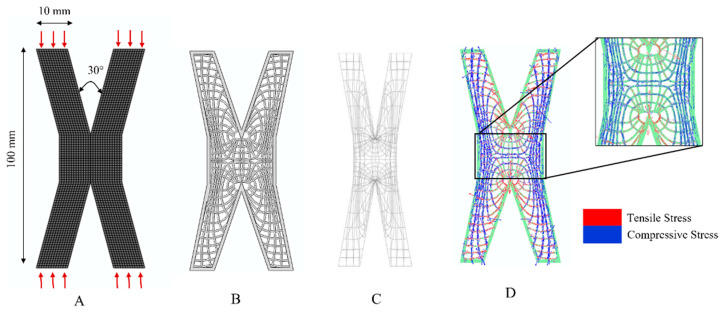
X-shaped model. (**A**) Finite element model of the X-shaped; (**B**) the principal stress trajectory of X-shaped; (**C**) the principal stress trajectory from Cowin; and (**D**) the principal stress distribution of porous structure.

## Data Availability

Not applicable.

## Appendix A

### Appendix A.1. Cantilever Beam Structure

As shown in Figure A1A, a two-dimensional cantilever beam finite element model with a rectangular of 50 × 20 mm was created. The bottom of the model was fixed and a force of 100N was applied to the center of the top. The material properties of the model were assigned based on the parameters of cortical bone. Due to only verifying the accuracy of the method, there are no strict requirements for the magnitude of the applied force. Figure A1B shows the porous structure based on the principal stress trajectory. Figure A1C shows the distribution of principal stress trajectory under the same boundary conditions as described in the literature [29]. Figure A1D shows the vector distribution of the principal stress field for the porous structure (Figure A1B) under the same loading conditions. In comparison, it is found that the distribution of the principal stress trajectory was essentially consistent with the results in the literature. The principal stress direction of the porous structure based on the principal stress trajectory was aligned with the truss direction of the porous structure.

### Appendix A.2. X-Shaped Structure

As shown in the Figure A2A, an X-shaped structure with an angle of 30° was created and axial loads were applied on the upper and lower surfaces. The distribution of principal stress trajectory (Figure A2B) was obtained through the stress visualization method. Compared with the results in the literature (Figure A2C), the distribution of the principal stress trajectory extracted using this method was identical to that in the literature [34]. Similarly, the porous structure established based on the principal stress trajectory still followed the truss direction of the porous structure under the same load loading (Figure A2D). The significance of proposing this model is that the structure can be approximated as a representative volume unit of the bone trabecular structure.
